# Seroprevalence of AIH-related autoantibodies in patients with acute hepatitis E viral infection: a prospective case–control study in China

**DOI:** 10.1080/22221751.2020.1722759

**Published:** 2020-02-10

**Authors:** Jian Wu, Naizhou Guo, Lifei Zhu, Xueyan Zhang, Cunquan Xiong, Jun Liu, Yanping Xu, Jun Fan, Jiong Yu, Qiaoling Pan, Jinfeng Yang, Hanying Liang, Xiuyuan Jin, Sunyi Ye, Wei Wang, Chengyuan Liu, Jinrong Zhang, Gongqi Li, Bin Jiang, Hongcui Cao, Lanjuan Li

**Affiliations:** a State Key Laboratory for the Diagnosis and Treatment of Infectious Diseases, National Clinical Research Center for Infectious Diseases, The First Affiliated Hospital, College of Medicine, Zhejiang University, Hangzhou, People’s Republic of China; b Department of Laboratory Medicine, The First People’s Hospital of Yancheng City, Yancheng, People’s Republic of China; c Department of Public Health, Jiangsu Vocational College of Medicine, Yancheng, People’s Republic of China; d Department of Laboratory Medicine, The Fifth People’s Hospital of Wuxi, Affiliated to Jiangnan University, Wuxi, People’s Republic of China; e Department of Laboratory Medicine, The People’s Hospital of Dafeng City, Yancheng, People’s Republic of China; f Department of Clinical Laboratory, Linyi Traditional Hospital, Linyi, People’s Republic of China; g Department of Laboratory Medicine, The Central Blood Station of Yancheng City, Yancheng, People’s Republic of China; h Zhejiang Provincial Key Laboratory for Diagnosis and Treatment of Aging and Physic-chemical Injury Diseases, Hangzhou, People’s Republic of China

**Keywords:** anti-nuclear antibody (ANA), anti-smooth muscle antibody (SMA), autoimmune hepatitis (AIH), acute hepatitis E (AHE), seroprevalence

## Abstract

The seroprevalenc of autoimmune hepatitis (AIH)-related antibodies in patients, particularly Asians, with acute hepatitis E (AHE) is unclear. In this study, we investigated whether acute hepatitis E virus (HEV) infection is associated with the seroprevalence of AIH-related autoantibodies and assessed their impact on the disease characteristics. AIH-related autoantibodies were detected by indirect immunofluorescence in 198 AHE patients and 50 type 1 AIH patients. The positivity rates of against nuclear antigen (ANA) and smooth muscles antibody (SMA) in AHE patients were 37.4% and 22.7%, and the total positivity rate was 50%. Compared to those in AIH patients, the positivity rates of ANA-H and SMA-AA were significantly lower (35.1% vs. 82.1% and 4.4% vs. 88.4%). Female gender and the ALT level, but not immunosuppressive or antiviral drugs, were independently predictive of the presence of AIH-related autoantibodies in AHE patients. Fifty-two patients positive for AIH-related autoantibodies were followed up for 12 months. During this period, 33 of them became negative and 19 remained positive, albeit with significantly decreased titres. In conclusions, the seroprevalence of AIH-related autoantibodies in AHE patients was elevated, particularly in females, but their subspecificities and titres differed from those of type 1 AIH. Acute HEV infection may be related to AIH.

**Abbreviations:** AIH: autoimmune hepatitis; AHE: acute hepatitis E; ANA: against nuclear antigen; SMA: smooth muscles antibody; ANA-H: ANA with homogeneous pattern; SMA-AA: SMA with anti-actin pattern; Anti-LKM1: anti- liver-kidney microsomes-1 antibody; ANCA: anti-neutrophil cytoplasmic antibody; AMA: anti-mitochondrial antibody; Anti-SLA: anti-soluble liver antigen; Anti-LC1: anti-liver cytoplasmic type 1 antibody; pANCA: perinuclear antineutrophil cytoplasmic antibody

## Introduction

Acute hepatitis E (AHE) is a viral hepatitis caused by hepatitis E virus (HEV) infection. HEV is transmitted mainly through faecal–oral routes and frequently causes major outbreaks or epidemics [1, 2]. Compared with other types of hepatitis, the mortality rate of AHE is high. AHE can cause severe jaundice and liver failure, leading to death. In the general population, the mortality rate of AHE is 1–4%, whereas in older people or pregnant women it can reach 20% [3]. AHE is concentrated in developing countries. However, the increasing global integration, development of transportation and tourism, frequent exchanges of people, and export of labour services has increased the prevalence of AHE in developed countries [4, 5]. Approximately one-third of the global population (∼2 billion) lives in HEV-epidemic areas, rendering AHE an important public-health issue globally [6]. The HEV has four major genotypes. Genotypes 1 and 2 are human viruses, an epidemiological pattern in most developing countries of the world, which are transmitted via faecal oral routes through contaminated water and then infect humans, causing many water-borne disease outbreaks [7]. The second group includes genotypes 3 and 4, which are zoonotic viruses that are common in pigs and humans, and have been associated with sporadic and limited food-borne outbreaks in developed parts of the world [8]. According to the World Health Organization (WHO), about 20 million people around the world are infected with HEV, 3.3 million of whom have symptoms, and about 60,000 people die of HEV related-liver failure every year [9]. In the past 10 years, the epidemiologic pattern in China has shifted from a pattern typical of developing areas to a pattern typical of developed countries [10]. Apart from sporadic HEV infection caused by HEV genotype 4, there are few large-scale outbreaks in China [11].

Autoimmune hepatitis (AIH) is a rare chronic inflammatory liver disease. It is characterized by elevated levels of aminotransferase and immunoglobulin G (IgG), female dominance, positivity for serum autoantibodies, moderate-to-severe interfacial hepatitis on histological examination, and a rapid response to steroid therapy [12, 13]. AIH is distributed globally in children and adults and can occur in any age group, but most frequently in those >40 years of age [14]. In view of the lack of population-based data, it is almost impossible to obtain accurate data for the prevalence of AIH. The average annual incidence rate of AIH-1 was estimated at 1.5 cases in Japan, 1.68 cases in Denmark, 2.0 cases in New Zealand and 3.0 cases in the United Kingdom per 100,000 individuals [15]. A nationwide epidemiological survey in Denmark showed that the annual incidence of AIH-1 was 1.68/100,000 and was increasing yearly [16]. AIH-2 mainly affects children and adolescents. An epidemiological study of 159 AIH children and adolescents in Canadian, showed that the annual incidence was 0.23 per 100,000 children [17]. Due to the high prevalence of chronic hepatitis B, few data are available on prevalence and incidence of AIH in South and East Asian countries [18]. Autoantibody testing plays an important role in the diagnosis of AIH–ANA and/or SMA for type 1 AIH and anti-liver-kidney microsomes-1 (LKM1) for type 2 AIH. Against nuclear antigen (ANA) and smooth muscles antibody (SMA) are antigenically heterogeneous, and each has subspecificities, such as ANA of homogeneous pattern (ANA-H) and anti-actin pattern (SMA-AA), which are associated with AIH [19, 20].

The aetiology of AIH is unclear, but it may be triggered by viruses. Buechter Matthias et al. [21] demonstrated that drugs, viral infections, and previous surgery may trigger acute liver failure (ALF) as the initial presentation of AIH. Navarta Luz et al. [22] reported that a relationship between hepatitis C virus (HCV) infection and the concurrent detection of various autoantibodies in the absence of symptoms of autoimmune diseases, and a link among the presence of a variety of autoantibodies simultaneously with SMA, increased Gamma-glutamyl transferase (GGT) levels and HCV titres in a population of male patients. Longhi et al. [23] found that up to 10% of HCV-infected individuals were positive for anti-LKM1, supporting the notion that HCV triggers type 2 AIH.

The relationship between HEV and AIH has been a focus of research. Eder et al. [24] evaluated anti-HEV antibodies in Austrian patients with AIH and found that anti-HEV, IgG-positive patients were older and had a serum prevalence almost twofold that of healthy Austrians. HEV may trigger AIH and cause outbreaks or recurrence later in the course of the disease. Pischke et al. [25] found that the serological infection rate of patients with AHE (7.7%) was higher than that of healthy blood donors (2%), patients with rheumatoid arthritis (3.5%), and patients with chronic hepatitis B and C infection (2.8%). However, van Gerven et al. [26] reported inconsistent conclusions, possibly due to the relatively high prevalence of HEV in The Netherlands. Notably, Terziroli Beretta-Piccoli Benedetta et al. [27] found that the positivity rate of AIH-related autoantibodies was significantly higher in patients with AHE, a few of whom developed AIH-specific autoantibodies. The virulence of HEV varies according to genotype. Therefore, we determined whether acute HEV infection in the Chinese population (genotype 4) is related to the presence of AIH-related autoantibodies.

## Patients and methods

### Patients

**Figure 1.**
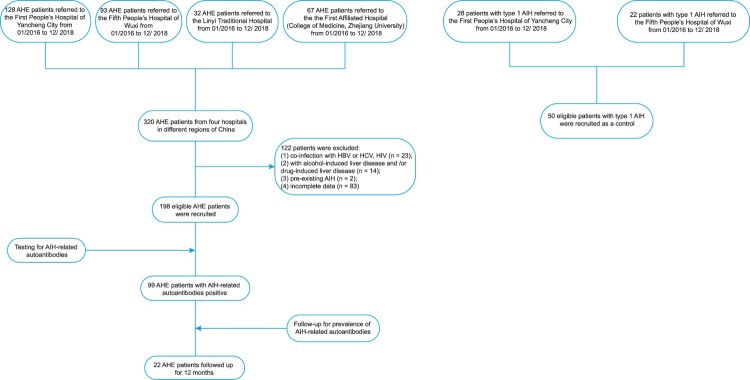
Screening of patients with AHE and type 1 AIH.

Sera from 198 patients with AHE who were referred to the First People’s Hospital of Yancheng City, the Fifth People’s Hospital of Wuxi, Linyi Traditional Hospital, and the First Affiliated Hospital (College of Medicine, Zhejiang University) from 1 January 2016 to 31 December 2018 were screened. Sera from 50 patients with type 1 AIH referred to the First People’s Hospital of Yancheng City or the Fifth People’s Hospital of Wuxi from 1 January 2016 to 31 December 2018 were also screened as controls. The protocol for patient enrolment is shown in Figure 1. All of the enrolled patients with AIH met the criteria of the International Autoimmune Hepatitis Group [28]. Samples were obtained at the time of diagnosis during the acute phase of disease before treatment and were stored at −80°C before analysis.

We evaluated the patients’ clinical and laboratory parameters, including age, sex, history of autoimmune disease, alcohol-induced liver disease, drug-induced liver disease, malignancy, liver fibrosis, immunosuppressive drugs, concomitant type 2 diabetes mellitus, other extrahepatic complications, history of exposure to patients with HEV, and alanine aminotransferase (ALT) and total bilirubin (TBIL) levels.

The inclusion criteria were as follows: diagnosis of HEV infection by reverse-transcription–polymerase chain reaction analysis of HEV RNA or anti-HEV immunoglobulin (Ig) M and by enzyme-linked immunosorbent assay (ELISA) for IgG (Wantai, Beijing, China). All patients had the typical profile of anti-HEV IgM positivity or a greater than twofold increase in the anti-HEV IgG titre. Hundred and thirty three of 198 patients were further confirmed by HEV RNA testing (Aikang, Hangzhou, China). The case of hepatitis E in this study was defined as positive serum anti-HEV IgM, and/or a greater than twofold increase in the anti-HEV IgG titre, and/or detectable HEV RNA with clinical presentation of acute hepatitis, which showed elevated liver enzymes and/or jaundice and/or non-specific symptoms such as fatigue, itching and nausea.

The exclusion criteria were as follows: (1) co-infection with hepatitis A virus (HAV), hepatitis B virus (HBV), HCV, or human immunodeficiency virus (HIV); (2) alcohol- and/or drug-induced liver disease; (3) pre-existing AIH; and (4) incomplete data.

Follow-up serum samples were collected 10–15 months later after diagnosis (median, 12 months). This study was performed in accordance with the Declaration of Helsinki and was approved by the local ethics committee. Written informed consent was obtained from all participants or their families.

### Testing for AIH-related autoantibodies

AIH-related autoantibodies were detected by indirect immunofluorescence (IIF) using rat kidney, liver, or stomach tissues (Euroimmun) [12]. HEp2 cells (Euroimmun) was performed to evaluate the patterns of AIH-related autoantibodies. The sera were analyzed by immunoblotting (Euroline Liver-Profile 4 IgG Euroimmun) for anti-soluble liver antigen, anti-liver cytosol type 1 (LC1), and anti-LKM1. The reactivity of anti-neutrophil cytoplasmic antibody (ANCA) was further analyzed using the ANCA Profile ELISA IgG (Euroimmun). The most suitable titration interval was provided by the dilution factor of 3.162 (square root of 10). Thus, the denominator of each step represents an integer power of 10, and the recommended minimum dilution is 1:100 (1:100, 1:320, 1:1000, 1:3200, 1:10,000, etc.).

### Statistical analysis

Statistical analyses were performed using the Statistical Package for the Social Sciences (SPSS) (v. 18.0; SPSS Inc., Chicago, IL). Univariate and multivariate logistic regression analyses were performed to identify independent predictors of positivity for AIH-related autoantibodies in patients with AHE. Categorical data are shown as numbers (percentages) and were compared by chi-squared test. A two-sided *P-*value < 0.05 was considered indicative of statistical significance.

## Result

### Patient characteristics

A total of 198 eligible patients with AHE were recruited from four hospitals in different regions of China. The majority of the patients with AHE were female 111 (56.1%), and 87 (43.9%) were male. The mean age of the patients was 55.3 years (range, 43.3–69.2 years). Of the 198 patients with AHE, none developed chronic infection. Of the 133 patients subjected to HEV RNA testing, 76 were positive. Of the 50 patients with Type 1 AIH as control, 42 (84.0%) were female and 8 (16.0%) were male. The mean age of the 50 patients with type 1 AIH was 56.7 years (range, 42.5–68.9 years). Among them, 26 (52.0%) were positive for both ANA and SMA, 13 (26.0%) for only ANA, and 11 (22.0%) for only SMA. The characteristics of AHE and type 1 AIH are shown in Table 1.

**Table 1.** Characteristics of the enrolled patients.

Variable	Type 1 AIH control group (*n* = 50)	AHE group (*n* = 198)	*P*
*Clinical characteristics*			
Age (y)	56.7 (42.50–68.90)	55.3 (43.31–69.20)	0.281
Females	84.0% (42/50)	56.1% (111/198)	0.0003
BMI	22.63 ± 2.27	23.21 ± 2.12	0.090
*Laboratory parameters*			
WBC (10^9^/L)	6.22 (5.50–8.58)	6.37 (5.59–8.96)	0.087
RBC (10^12^/L)	4.20 ± 0.67	4.15 ± 0.72	0.657
ALT (U/L)	265.00 (122.00–875.00)	534.00 (139.00–1498.00)	0.001
AST (U/L)	329.00 (84.00–992.00)	377.00 (98.00–1190.00)	0.086
GGT (U/L)	95.00 (55.58–170.00)	99.00 (56.50–175.00)	0.198
TP (g/L)	58.76 ± 9.12	57.20 ± 8.12	0.238
ALB(g/L)	30.76 ± 5.61	31.39 ± 5.62	0.479
UREA (mmol/L)	4.51 (3.58–6.95)	4.59 (3.72–7.05)	0.365
CR (umol/L)	75.12 (63.55–92.50)	76.22 (64.55–95.60)	0.176
PT (s)	17.08 (14.02–21.09)	17.45 (14.22–23.55)	0.116
PLT (10^9^/L)	135.00 (89.00–168.00)	138.00 (91.00–174.00)	0.169
AFP (ng/ml)	33.41 (7.98–108.00)	37.20 (6.90–125.80)	0.217
TBIL (umol/L)	55.87 ± 9.32	295.57 ± 159.12	0.000
CHE (U/L)	3855.00 (2150.40–4916.90)	2728.50 (2345.52–3312.68)	0.001
CRP (mg/L)	9.89 (6.76–13.98)	10.22 (7.01–14.09)	0.082
INR	1.80 (1.35–2.32)	1.77 (1.29–2.15)	0.198
HEV-IgM (+)	0.0% (0/50)	80.3% (159/198)	<0.0001
HEV-IgG (+)	14.0% (7/50)	84.8% (168/198)	<0.0001
*Symptoms*			
Jaundice	4.0% (2/50)	71.2% (141/198)	<0.0001
Fever	2.0% (1/50)	57.1% (113/198)	<0.0001
Nausea/vomit	14.0% (7/50)	70.2% (139/198)	<0.0001
Abdominal pain	20.0% (10/50)	24.2% (48/198)	0.527

Note: AST, Glutamic oxaloacetic transaminase; ALT, Alanine aminotransferase; AFP, Alpha fetoprotein; WBC, white blood cell; RBC, red blood count; CHE, cholinesterase; UREA, urea nitrogen; CR, creatinine; PT, prothrombin time; TBIL, total bilirubin; ALB, albumin; INR, international normalized ratio; PLT, platelet; CRP, c-reactive protein.

Of the 198 patients with AHE, 9 were taking immunosuppressive drugs. Of these, there were two patients with hyperthyroidism (methylprednisolone 500 mg once daily for 3 days continuously for 1 month per cycle; methylprednisolone 500 mg once daily for 3 days continuously for 1 week per cycle), two with aplastic anaemia (cyclosporin 3–5 mg/kg daily continuously), one with chronic lymphocytic leukaemia (fludarabine 25 mg twice daily for five consecutive days), one with sarcoidosis (prednisone 20 mg/day), one with nephrotic syndrome (prednisone, 30 mg/day), one with myasthenia gravis (methylprednisolone 500 mg once daily for 5 days, followed by 240 or 120 mg daily for 5 days and, finally, 60 mg methylprednisolone continuously), and one patient with Guillain–Barre syndrome (methylprednisolone 500 mg once daily for 5 days).

Of the 198 patients with AHE, 10 had past or current autoimmune disease. Three patients had type I diabetes, two had myasthenia gravis, and one each had giant-cell arteritis, Guillain–Barre syndrome, systemic sarcoidosis, rheumatism, and Hashimoto thyroiditis.

### Prevalence of AIH-related autoantibodies

The prevalence and subspecificities of serum AIH-related autoantibodies in the patients with AHE are shown in Table 2. The serum of 74 patients (37.4%) was positive for ANA, which covering with the vast majority of cases, mainly of a non-H pattern (48 of 74 [64.9%]). SMA was detected in the sera of 45 patients (22.7%), among whom 43 (95.6%) exhibited non-anti-actin specificity. The serum of 32 patients (16.2%) was positive for ANCA, all of whom had atypical perinuclear antineutrophil cytoplasmic antibody (pANCA). All of the patients were negative for AMA, anti-soluble liver antigen, anti-LKM1, and anti-LC1. Because the 32 ANCA-positive patients had atypical pANCA and were positive for ANA, subsequent experiments focused on ANA and SMA. Of note, 20 patients (10.1%) were positive for ANA and SMA antibodies. Thus, 99 of the 198 patients with AHE (50%) were positive for AIH-related autoantibodies: 73 (36.9%) for ANA non-H and/or SMA non-AA and 26 (13.1%) for ANA-H or SMA-AA.

**Table 2.** Serum AIH-related autoantibodies in 198 AHE patients.

AIH-related autoantibodies	Positive patients
ANA	74/198 (37.4%)
ANA-H	26/198 (13.1%)
Other ANA	48/198 (24.3%)
SMA	45/198 (22.7%)
SMA-AA	2/198 (1.0%)
Other SMA	43/198 (21.7%)
ANCA	32/198 (16.2)
Atypical pANCA	32/198 (16.2%)
ANA-H + SMA-AA	2/198 (1.0%)
Other ANA + Other SMA	18/198 (9.1%)
AMA, anti-SLA, anti-LKM1 and anti-LC1	0/198 (0.0%)

Note: AHE, Acute hepatitis E; ANA, Against nuclear antigen; SMA, Smooth muscles antibody; ANA-H, ANA with homogeneous pattern; SMA-AA, SMA with anti-actin pattern; Anti-LKM1, Anti- liver-kidney microsomes-1 antibody; ANCA, Anti-neutrophil cytoplasmic antibody; pANCA, perinuclear antineutrophil cytoplasmic antibody; AMA, Anti-mitochondrial antibody; Anti-SLA, Anti-soluble liver antigen; Anti-LC1, Anti-liver cytoplasmic type 1 antibody.

**Figure 2.**
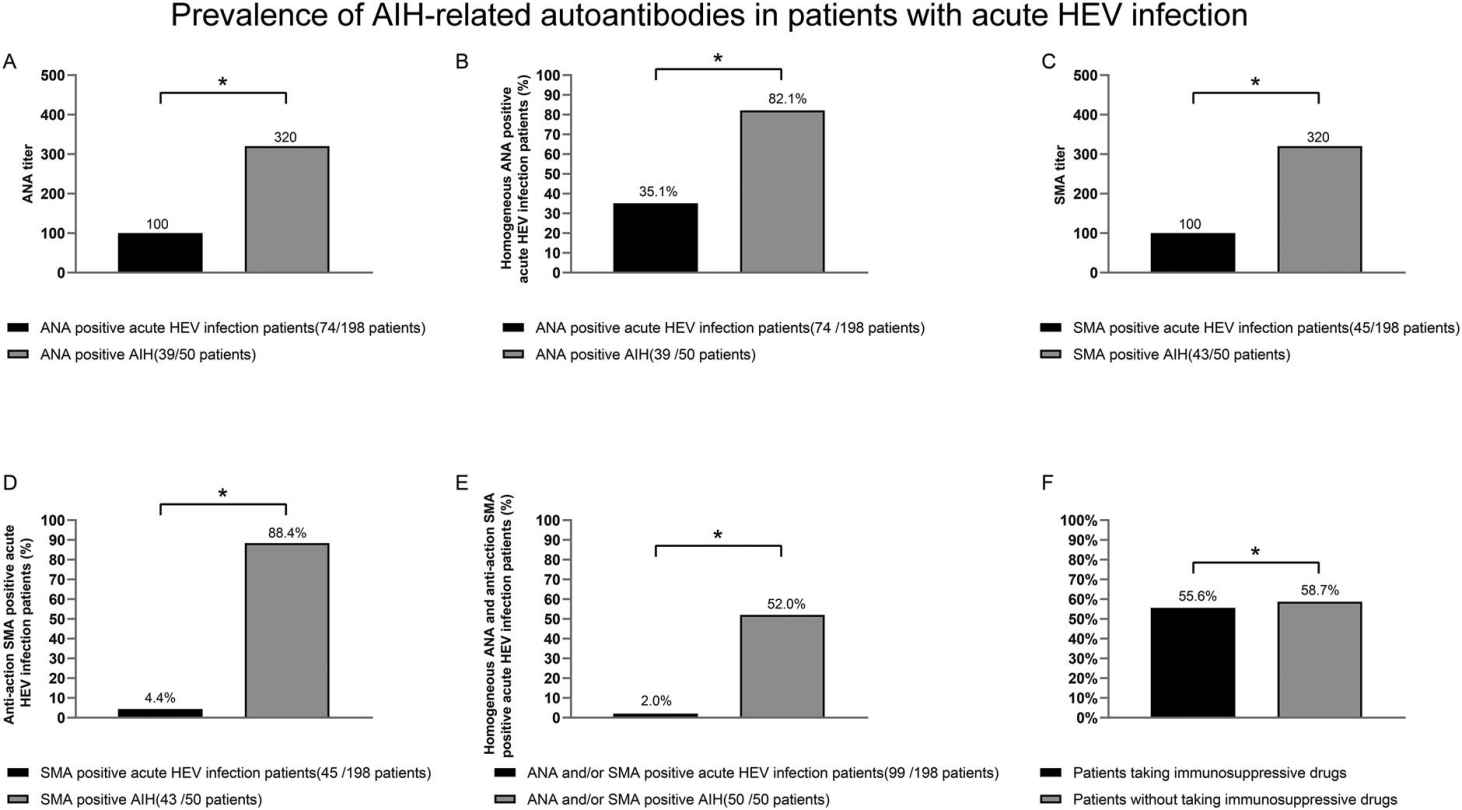
Prevalence of AIH-related autoantibodies in patients with AHE. *P < 0.05. Titres of ANA and SMA (A, C), prevalence of ANA-H and SMA-AA (B, D), and simultaneous positivity for ANA-H and SMA-AA (E) in patients with AHE and those with type 1 AIH. Positivity for AIH-related autoantibodies in AHE patients according to the use of immunosuppressive drugs (F). ANA, against nuclear antigen; SMA, smooth muscle actin; ANA-H, ANA of homogeneous pattern; SMA-AA, SMA of anti-actin pattern.

Compared with the 50 patients with AIH, the prevalence of ANA-H and SMA-AA were lower in the patients with AHE (35.1% vs. 82.1%, *P *< 0.05; and 4.4% vs. 88.4%, *P* < 0.05, respectively); the ANA and SMA titres were also lower (both 1:320 vs. 1:1000, *P* < 0.05). Moreover, the rate of simultaneous positivity for ANA-H and SMA-AA was significantly lower than that in the patients with AIH (2.0% vs. 52.0%, *P* < 0.05) (Figure 2(A–E)). There was no difference in the rate of positivity for AIH-related autoantibodies in the patients with AHE according to the use of immunosuppressive drugs (Figure 2(F)).

### Correlations between AIH-related autoantibodies and clinicopathological parameters

The 198 patients were divided into those positive and those negative for AIH-related autoantibodies. Univariate analyses showed that patients positive for AIH-related autoantibodies were more frequently female and had a higher international normalized ratio and ALT, aspartate aminotransferase (AST), and IgG levels. The other parameters (past or current autoimmunity, antiviral therapy, jaundice, and immunosuppressive drugs) did not differ significantly between the two groups. In a multivariate analysis, female sex and the ALT level were independent risk factors for positivity for AIH-related autoantibodies in patients with AHE (Table 3).

**Table 3.** Characteristics of the AIH-related autoantibodies positive and negative AHE patients.

Variable	AIH-related autoantibodies positive group (*n* = 99)	AIH-related autoantibodies negative group (*n* = 99)	*P*	Univariate analysis		Multivariate analysis	
				OR (95% CI)	*P*	OR (95% CI)	*P*
Age (y)	55.21 ± 11.52	56.18 ± 12.47	0.102	1.00 (0.98–1.05)	0.891		
Females	66.7% (66/99)	45.5% (45/99)	0.021	0.94 (0.39–4.22)	<0.001	1.02 (0.42–5.44)	0.003
WBC (10^9^/L)	6.42 (5.51–8.88)	6.21 (5.64–8.87)	0.443	2.27 (0.96–5.25)	0.879		
RBC (10^12^/L)	4.18 ± 0.65	4.09 ± 0.61	0.551	1.09 (0.81–1.34)	0.477		
ALT (U/L)	592.00 (147.00–1381.00)	375.00 (104.00–915.00)	0.007	0.75 (0.61–0.98)	<0.001	0.79 (0.67–1.08)	0.012
AST (U/L)	382.00 (95.00–1105.00)	208.00 (81.00–618.00)	0.032	0.74 (0.62–0.93)	0.002		
GGT (U/L)	99.00 (57.50–172.00)	98.00 (57.00–175.50)	0.513	0.46 (0.29–0.79)	0.533		
TP (g/L)	57.54 ± 8.25	56.56 ± 9.16	0.659	0.97 (0.94–1.01)	0.285		
ALB (g/L)	31.59 ± 5.62	31.31 ± 5.67	0.978	0.95 (0.88–0.97)	0.068		
UREA (mmol/L)	4.56 (3.50–6.97)	4.62 (3.78–7.02)	0.553	2.10 (1.35–4.64)	0.098		
CR (umol/L)	76.43 (66.50–93.00)	76.00 (65.50–93.50)	0.550	1.69 (0.79–3.71)	0.170		
PT (s)	17.39 (15.03–23.12)	17.47 (14.95–22.19)	0.679	5.71 (2.65–19.76)	0.096		
INR	1.97 (1.92–2.29)	1.51 (1.22–2.02)	0.043	6.94 (2.55–16.18)	0.009		
PLT (10^9^/L)	136.00 (90.50–169.00)	139.00 (92.00–172.50)	0.553	0.52 (0.27–0.89)	0.059		
CRP (mg/L)	10.99 (7.15–14.25)	9.56 (7.03–13.89)	0.986	0.98 (0.97–1.16)	0.977		
TBIL (umol/L)	296.27 ± 163.12	290.17 ± 155.42	0.932	1.01 (1.00–1.01)	0.014		
AFP (ng/ml)	35.41 (7.20–115.00)	38.10 (6.90–122.20)	0.905	0.99 (0.81–1.23)	0.953		
CHE (U/L)	2795.80 (2353.60–3155.80)	2688.80 (2418.35–3212.77)	0.337	0.98 (0.22–0.69)	0.655		
HEV-IgG	85.9% (85/99)	83.8% (83/99)	0.665	1.22 (0.32–0.89)	0.499		
Jaundice	74.7% (74/99)	67.7% (67/99)	0.844	1.31 (0.35–0.81)	0.505		
IgG (mg/dL)	2.22 ± 0.55	1.50 ± 0.41	0.028	0.68 (0.52–1.88)	0.005		
Taking immuno-suppressive drugs	5.1% (5/99)	4.0% (4/99)	0.905	1.43 (0.19–0.79)	0.662		
Past or current autoimmune diseases	6.1% (6/99)	4.0% (4/99)	0.337	1.33 (0.23–0.91)	0.506		
Antiviral therapy	79.8% (79/99)	72.7% (72/99)	0.962	1.09 (0.36–0.94)	0.294		

Note: AST, Glutamic oxaloacetic transaminase; ALT, Alanine aminotransferase; AFP, Alpha fetoprotein; WBC, white blood cell; RBC, red blood count; CHE, cholinesterase; UREA, urea nitrogen; CR, creatinine; PT, prothrombin time; TBIL, total bilirubin; ALB, albumin; INR, international normalized ratio; PLT, platelet; CRP, c-reactive protein.

Subsequently, according to their AIH-related autoantibody status, we divided the 198 AHE patients into the following three groups: negative for AIH-related antibodies (group 1), positive for other ANA and/or SMA (group 2), and positive for ANA-H and/or SMA-AA (group 3) (Table 4). The levels of ALT, c-reactive protein (CRP), and IgG increased slightly from group 1 to group 3 (*P* < 0.05 or *P* < 0.01). Univariate analyses showed that the proportion of females and the levels of ALT and IgG were significantly higher in group 3 than in groups 1 and 2 (all *P* < 0.05). A multivariate logistic regression analysis revealed that female sex and the ALT level were independent predictors of the presence of AIH-related antibodies in AHE patients (both *P* < 0.05).

**Table 4.** Characteristics of the AIH-related autoantibodies different status in AHE patients.

Variable	Group1 Autoantibody negative (*n* = 99)	Group 2 Other ANA and/or SMA (*n* = 73)	Group 3 ANA-H or SMA-AA (*n* = 26)	Univariate analysis		Multivariate analysis	
				OR (95% CI)	*P*	OR (95% CI)	*P*
Age (y)	56.18 ± 12.47	55.17 ± 11.09	56.82 ± 12.45	1.01 (0.97–1.04)	0.825		
Gender (F/M)	45.5% (45/54)	60.3% (44/29)*	84.6% (22/4)**	0.89 (0.39–4.22)	<0.001	1.03 (0.42–5.26)	0.002
WBC (10^9^/L)	6.21 (5.64–8.87)	6.34 (5.69–8.28)	6.46 (5.48–8.90)	2.12 (0.96–5.63)	0.917		
RBC (10^12^/L)	4.09 ± 0.61	4.19 ± 0.55	4.09 ± 0.59	1.10 (0.83–1.29)	0.466		
ALT (U/L)	375.00 (104.00–915.00)	464.00 (145.00–1209.00)*	639.00 (156.00–1408.00)**	0.75 (0.59–0.90)	0.001	0.79 (0.65–0.98)	0.021
AST (U/L)	208.00 (81.00–618.00)	368.00 (95.00–1105.00)	409.00 (83.00–908.00)	0.95 (0.69–1.23)	0.433		
GGT (U/L)	98.00 (57.00–175.50)	99.50 (57.00–172.50)	98.30 (56.50–175.00)	1.08 (0.38–1.25)	0.262		
TP (g/L)	56.56 ± 9.16	57.94 ± 8.11	56.22 ± 9.05	0.97 (0.93–1.09)	0.299		
ALB (g/L)	31.31 ± 5.67	31.98 ± 5.09	31.03 ± 4.96	1.09 (0.87–1.11)	0.133		
UREA (mmol/L)	4.62 (3.78–7.02)	4.44 (3.21–6.88)	4.67 (3.79–7.25)	2.99 (1.21–4.98)	0.187		
CR (umol/L)	76.00 (65.50–93.50)	76.95 (69.50–98.50)	76.06 (65.00–94.50)	1.89 (0.80–4.79)	0.269		
PT (s)	17.47 (14.95–22.19)	17.10 (14.13–21.10)	17.55 (14.80–23.60)	4.67 (2.43–20.31)	0.098		
INR	1.51 (1.22–2.02)	1.98 (1.92–2.29)*	1.88 (1.32–2.16)	3.29 (2.59–11.45)	0.076		
PLT (10^9^/L)	139.00 (92.00–172.50)	134.00 (92.00–168.00)	139.00 (95.00–189.00)	0.59 (0.29–1.02)	0.068		
CRP (mg/L)	9.56 (7.03–13.89)	10.91 (7.19–14.96)*	12.07 (7.21–15.22)**	1.02 (0.87–1.79)	0.933		
TBIL (umol/L)	290.17 ± 155.42	292.12 ± 160.55	301.09 ± 169.12	1.29 (0.98–1.12)	0.104		
AFP (ng/ml)	38.10 (6.90–122.20)	35.09 (7.10–98.00)	36.30 (7.90–120.20)	0.97 (0.85–1.29)	0.829		
CHE (U/L)	2688.80 (2418.35–3212.77)	2905.80 (2260.33–3198.80)	2708.50 (2478.92–3055.89)	1.02 (0.41–1.03)	0.667		
HEV-IgG	83.8% (83/99)	86.3% (63/73)	84.6% (22/26)	1.19 (0.30–0.91)	0.698		
Jaundice	67.7% (67/99)	74.0% (54/73)	76.9% (20/26)	1.39 (0.34–0.85)	0.588		
IgG (mg/dL)	1.50 ± 0.41	2.21 ± 0.46*	2.98 ± 0.59**	0.62 (0.50–1.68)	0.005		
Taking immuno-suppressive drugs	4.0% (4/99)	5.5% (4/73)	3.8% (1/26)	1.49 (0.19–0.88)	0.677		
Past or current autoimmune diseases	4.0% (4/99)	5.5% (4/73)	7.7% (2/26)	1.33 (0.23–0.91)	0.506		
Antiviral therapy	72.7% (72/99)	79.5% (58/73)	80.8% (21/26)	1.13 (0.35–0.99)	0.334		

Notes: AST, Glutamic oxaloacetic transaminase; ALT, Alanine aminotransferase; AFP, Alpha fetoprotein; WBC, white blood cell; RBC, red blood count; CHE, cholinesterase; UREA, urea nitrogen; CR, creatinine; PT, prothrombin time; TBIL, total bilirubin; ALB, albumin; INR, international normalized ratio; PLT, platelet; CRP, c-reactive protein; ANA, Against nuclear antigen; SMA, Smooth muscles antibody; ANA-H, ANA with homogeneous pattern; SMA-AA, SMA with anti-actin pattern.*P < 0.05; **P < 0.01.

### Case presentations

**Figure 3.**
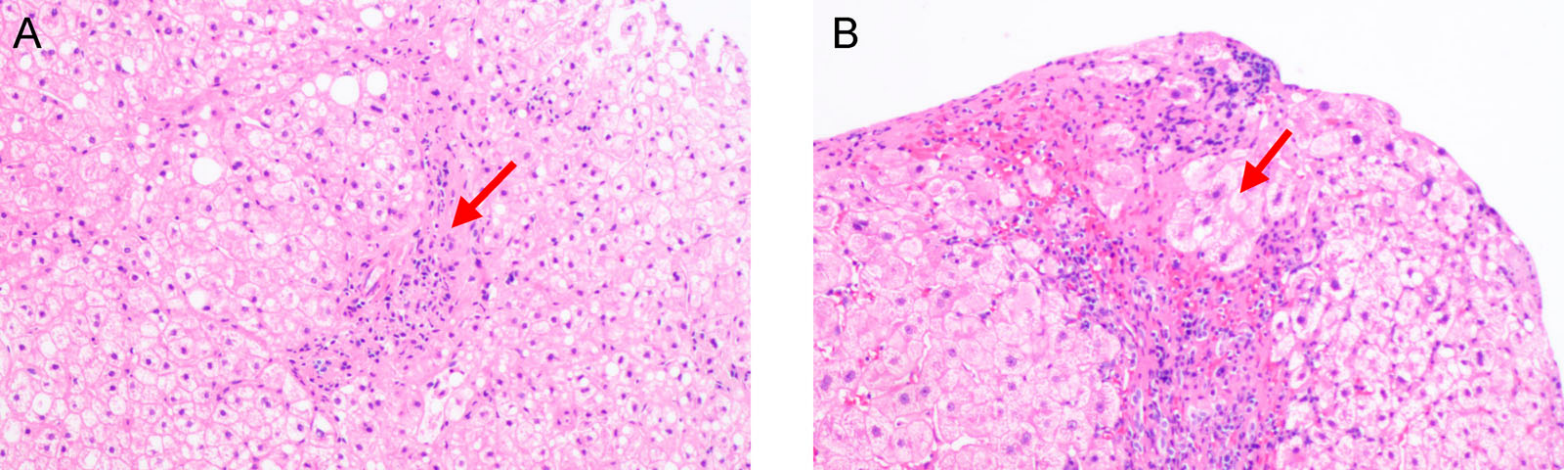
Histological examination demonstrated the presence of interfacial hepatitis (A) and rosettes of hepatocytes (B).

An 83-year-old male presented with severe yellowish skin and sclera, general fatigue for 1 week, oedema of both lower limbs, and positive shifting dullness. There was no history of hypertension, diabetes, coronary heart disease, or cerebral infarction. The patient had operated spray-door surgery one year prior, when routine detection found that the levels of immunoglobulin, erythrocyte sedimentation rate (ESR) and complement were normal. Liver function tests showed that the levels of ALT, AST and TBIL were normal. Tests for HAV, HBV, HCV and HEV showed negative results. AIH-related autoantibodies yielded positive results for ANA-H (titre 1:320) and SMA-AA (titre 1:320). During the patient’s routine examination, liver function tests showed an ALT level of 520 U/L, an AST level of 346.2 U/L, and a TBIL level of 157.52 µmol/L. The level of IgG and IgM were 35 and 2.85 g/L respectively. The level of complement 3 (C3) and complement 4 (C4) were 0.55 and 0.08 g/L respectively. A blood coagulation function test showed that the prothrombin time was 26.5 s. Haematological examination showed that the white blood cell count and CRP level were increased. The level of ESR was 55 mm/h. The patient’s serum was positive for anti-HEV IgM and IgG but negative for HEV RNA. Tests for HAV, HBV, HCV, HIV and *Treponema pallidum* (TP) yielded negative results. Tests for AIH-related autoantibodies yielded positive results for ANA-H (titre 1:320) and SMA-AA (titre 1:100). Histological examination demonstrated the presence of interfacial hepatitis and rosettes of hepatocytes (Figure 3). The final diagnosis was AHE with AIH. After hepatoprotective treatment with glycyrrhizic acid and acetylcysteine, the transaminase and bilirubin levels normalized rapidly, and the jaundice subsided. The levels of IgG, ESR were normal, while C3 and C4 were near normal. At the 4-month follow-up, the patient’s serum was negative for anti-HEV IgM but positive for anti-HEV IgG. At this time, he was also positive for ANA and SMA, albeit with significantly decreased titres.

### Follow-up of the prevalence of AIH-related autoantibodies

The 99 patients positive for AIH-related antibodies were followed up, with 52 followed up for 12 months. The ALT level of 98 patients rapidly recovered to within the normal range, and one elderly patient died of liver failure after 2 weeks. None of the 52 patients followed up for 12 months were re-admitted to the hospital for persistent liver disease. Among the 52 patients, 33 were negative for AIH-related antibodies, and 19 were positive but with a lower titre. No new serological positivity was found in the 52 patients during the 12-month follow-up.

## Discussion

Several studies of the role of hepatitis viruses in AIH are extant. Abdel-Ghaffar et al. [29] detected AIH-related antibodies in 63% of children with acute HAV infection, which was significantly higher than the rate in healthy children. Vento et al. [30] conducted a 4-year prospective study of 58 relatives of 13 AIH patients. Three of the patients had sub-clinical HAV infection, two of whom developed type 1 AIH within 5 months, suggesting defective inhibition of the T cell-mediated immune response to hepatic antigens. Gregorio et al. [31] evaluated 61 children with HBV treated with interferon and found that 42 (69%) were positive for AIH-related antibodies, confirming that AIH-related antibodies are frequently present in patients with chronic HBV infection. Cassani et al. [32] assessed the relationship between hepatitis C virus and AIH; the positivity rates of ANA, SMA, and anti-LKM1 in patients with chronic hepatitis C were 9%, 20%, and 6%, respectively. However, their subspecificity was different from that of patients with AIH; i.e. SMA was predominant.

Tatsuo [33] and Luzdivina [34] reported that patients who developed AIH after AHE were positive for ANA (but did not describe the type pattern) and had a hepatic histology typical of AIH. Terziroli Beretta-Piccoli Benedetta et al. [27] evaluated AIH-related antibodies in 50 patients with acute HEV infection. More than 50% were positive for AIH-related antibodies; five patients had the ANA-H pattern and two had the SMA-AA pattern.

In this study, we evaluated 198 Chinese patients with AHE and 50 patients with typical type 1 AIH as controls. We investigated the (1) seroprevalence of the ANA-H and SMA-AA patterns, (2) antibody titres, (3) and the seroprevalence of both ANA-H and SMA-AA positive. Of the 198 patients with AHE, 74 (37.4%) were positive for ANA, of whom 26 (35.1%) had the ANA-H pattern; 45 (22.7%) were positive for SMA, of whom 2 (4.4%) had the SMA-AA pattern; and 32 (16.2%) were positive for ANCA, all of whom were atypical. After excluding 20 patients with both ANA and SMA, the total positivity rate of AIH-related antibodies was 50% (99/198). The titres of HEV-related ANA and SMA were significantly lower than those of patients with type 1 AIH, and the positivity rates of ANA-H and SMA-AA were significantly different. Also, only 2 of 198 patients with HEV were positive for both ANA-H and SMA-AA. Unlike prior studies, ANA was the predominant HEV-related autoantibody, and most had ANA with speckles.

We assessed the correlation between AIH-related antibodies and the clinical parameters of the patients with AHE. In line with previous reports, AIH-related antibodies were related to sex. Most of the patients positive for AIH-related antibodies were female and showed high biochemical and histological activity, suggesting that female-related autoimmune responses aggravate liver disease. Notably, when the patients were grouped according to their AIH-related antibody status, the ALT and IgG levels gradually increased related to the type of self-activity existing, reaching the highest value. Multivariate In a multivariate logistic regression analysis, female sex and the ALT level, but not immunosuppressive or antiviral drugs, were independent predictors of the presence of AIH-related antibodies in patients with AHE.

Encouragingly, two patients positive for both ANA-H and SMA-AA were eventually diagnosed with type 1 AIH based on the results of AIH-related antibody testing and histopathological examination. Due to the data for one of them was not sufficient, we only reported details regarding the admission, diagnosis, treatment, and recovery of other case. After hepatoprotective treatment, the levels of ALT, TBIL, IgG and ESR for the patient recovered rapidly to within the normal ranges. After 4 months of follow-up, the patient’s serum was negative for anti-HEV IgM but positive for anti-HEV IgG, and AIH-related antibodies had significantly decreased titres.

HEV usually causes asymptomatic and self-limiting disease [35, 36]. After antiviral treatment, one elderly patient died of HEV-related ALF; the other 98 patients with AHE recovered rapidly. We followed-up 52 patients for 12 months, of whom 33 became negative, and 19 remained positive for AIH-related antibodies, albeit with significantly decreased titres. No new antibodies were detected in serum after treatment.

This study had several limitations. First, the 198 patients with AHE were enrolled from four hospitals in different regions of China; therefore, the study may have been subject to selection bias. Second, although HEV genotype 4 is predominant in China, we did not determine the HEV genotype. Third, HEV RNA was not assessed in all of the patients, so the correlations between HEV RNA and AIH-related antibodies were not discussed. Fourth, it is very interesting and important to investigate whether autoantibodies are produced during follow-up in seropositive patients with acute infection; however, we are failure to follow up the negative serum in this study.

In summary, this study yielded the following three findings. First, the seroprevalence of AIH-related autoantibodies in patients with AHE was elevated, particularly in females, but their subspecificities and titres, as well as the rate of type 1 AIH were different compared with typical type 1 AIH patients. Second, non-specific AIH-related autoantibodies are often produced during acute HEV infection, which should thus be excluded when diagnosing AIH. Immunosuppression can lead to chronic HEV disease and has implications for HEV diagnosis, treatment, and prognosis. Third, acute HEV infection may be related to AIH, but it needs further study. Therefore, long-term follow-up of the seroprevalence of AIH-related autoantibodies is needed.
